# Evolution and Function of Dinosaur Teeth at Ultramicrostructural Level Revealed Using Synchrotron Transmission X-ray Microscopy

**DOI:** 10.1038/srep15202

**Published:** 2015-10-29

**Authors:** Chun-Chieh Wang, Yen-Fang Song, Sheng-Rong Song, Qiang Ji, Cheng-Cheng Chiang, Qingjin Meng, Haibing Li, Kiko Hsiao, Yi-Chia Lu, Bor-Yuan Shew, Timothy Huang, Robert R. Reisz

**Affiliations:** 1 National Synchrotron Radiation Research Center, 30076 Hsinchu, Taiwan; 2 Department of Geosciences, National Taiwan University, 10617 Taipei, Taiwan; 3 Institute of Geology, Chinese Academy of Geological Sciences, 100037 Beijing, China; 4 Beijing Museum of Natural History, 100050 Beijing, China; 5 Department of Geology, Chinese Culture University, 11114 Taipei, Taiwan; 6 Institute of Genomics and Bioinformatics, National Chung Hsing University, 402 Taichung, Taiwan; 7 Department of Biology, University of Toronto Mississauga, Mississauga, Ontario L5L 1C6, Canada; 8 Department of Optics and Photonics, National Central University, 32001 Tauyuan, Taiwan

## Abstract

The relationship between tooth form and dietary preference is a crucial issue in vertebrate evolution. However, the mechanical properties of a tooth are influenced not only by its shape but also by its internal structure. Here, we use synchrotron transmission X-ray microscopy to examine the internal microstructures of multiple dinosaur teeth within a phylogenetic framework. We found that the internal microstructures of saurischian teeth are very different from advanced ornithischian teeth, reflecting differences in dental developmental strategies. The three-tissue composition (enamel–mantle dentin–bulk dentin) near the dentinoenamel junction (DEJ) in saurischian teeth represents the primitive condition of dinosaur teeth. Mantle dentin, greatly reduced or absent from DEJ in derived ornithischian teeth, is a key difference between Saurischia and Ornithischia. This may be related to the derived herbivorous feeding behavior of ornithischians, but interestingly, it is still retained in the herbivorous saurischian sauropods. The protective functions of mantle dentin with porous microstructures between enamel and bulk dentin inside typical saurischian teeth are also discussed using finite-element analysis method. Evolution of the dental modifications in ornithischian dinosaurs, with the absence of mantle dentin, may be related to changes in enamel characteristics with enamel spindles extending through the DEJ.

Ancient vertebrate teeth command great interest among paleontologists, as they are well preserved, abundant, and easily available. Such teeth provide information about the feeding habits^1^,^2^, ages^3^, habitats and environments^4^, food resources^5^, and evolution of jawed vertebrates^5^,^6^. Although morphological description is the usual method of identifying, classifying, and studying the function of fossil teeth^7^,^8^,^9^,^10^, scientists have recently applied new analytical techniques for gaining greater insights into their internal structure. For example, Hwang^11^,^12^,^13^ proposed using scanning electron microscopy (SEM) to diagnose dinosaur taxa based on the diversity of enamel microstructures, which are an excellent reference for studying evolutionary trends within dinosaurs.

To date, however, the internal fine structures of dinosaur teeth have not been thoroughly studied because of limitations in analytical techniques. For example, X-ray micro-computed tomography (μ-CT) can provide three-dimensional (3D) information on the internal microstructures of teeth non-destructively^14^,^15^, but cannot reveal internal structures at scales smaller than 1 μm. When combined with focused ion beam (FIB) techniques, however, SEM-FIB can provide 3D internal information on specimens at a nanometer-level spatial resolution^16^, but these are destructive and time-consuming methods. In this study, we use synchrotron transmission X-ray microscopy (TXM)^17^,^18^ to identify the 3D ultramicrostructures of various kinds of dinosaur teeth. TXM employs a Fresnel zone plate objective to achieve a spatial resolution of up to 30 nm^19^, along with ultrahigh-brightness synchrotron hard X-rays. This permits investigations into the diversity of 3D internal ultramicrostructures of dinosaur teeth from both saurischian and ornithischian dinosaurs and explore the evolutionary trends of these dinosaurs.

## Results

**Figure 1:**
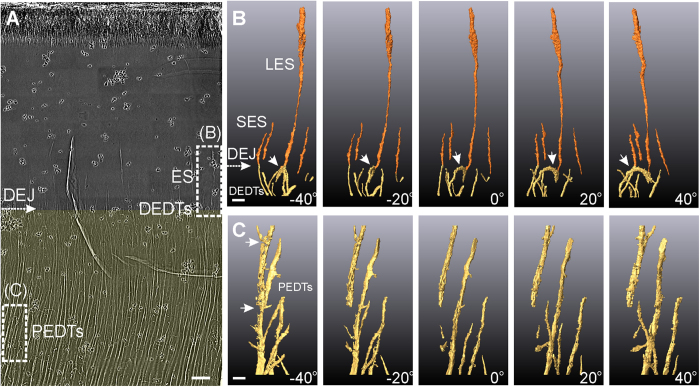
Ultrahigh-resolution internal hollow microstructures of a *Saurolophus* tooth obtained via TXM. (**A**) 2D X-ray photograph of a sectioned *Saurolophus* tooth. White-dashed rectangles indicate the 3D reconstruction regions shown in (**B**,**C**), with the viewing angles normal to incident X-rays at −40°, −20°, 0°, 20°, and 40°. Dentin is colored translucent yellow, and enamel is uncolored. (Scale bar: 10 μm.) (**B**) Long and short enamel spindles (LES and SES, in orange) and distal ends of the dentinal tubules (DEDTs, in yellow). The bulbous LES and small SES are approximately 30 μm and 10 μm in length, respectively. White arrows indicate a curved dentinal tubule (DT) near the dentinoenamel junction (DEJ). (Scale bar: 2 μm.) (**C**) Proximal ends of the dentinal tubules (PEDTs), which are approximately 70 μm away from the DEJ. White arrows indicate structural defects in the DTs. (Scale bar: 2 μm.) (The small black dots in (**A**) are gold particles, which are used for system and image positioning).

Figure 1 shows the internal tooth microstructures of *Saurolophus,* a Late Cretaceous ornithischian dinosaur. This figure displays two significant types of internal hollow microstructures: enamel spindles (ES) and dentinal tubules. Figure 1B,C show the 3D reconstructed hollow microstructures of the dashed rectangular regions of Fig. 1A, comprising the ESs (see Fig. 1B and Supplementary Movie S1), the distal end of the dentinal tubules (DEDTs, Fig. 1B), the proximal end of the dentinal tubules (PEDTs, Fig. 1C), and dentinoenamel junction (DEJ, Fig. 1A,B).

ESs are caused by odontoblasts extending into enamel matrices during amelogenesis^20^. We recognize two particular types of ESs: long enamel spindles (LES) with large diameters (approximately 600 nm at the terminal end) and short enamel spindles (SES) with small diameters (approximately 100 nm). Some of the LESs originated from the combination of multi-dentinal-tubule extensions (see Supplementary Fig. S1 and Supplementary Movie S2), and this can be clearly detected using 3D tomography.

Dentinal tubules are crucial fine structures in teeth, and have been intensely studied in human dental histology^21^,^22^,^23^ and vertebrate paleontology^6^. The complex 3D organization of dentinal tubules influences metabolism, signal transduction^24^, and the mechanical characteristics of teeth^21^,^22^. Parkinson^21^ and Zaslansky^22^ used projection-based X-ray μ-CT methods to perform 3D reconstructions of dentinal tubules in human teeth. However, these methods could not show the details of dendritic tubular structures, as they were constrained by their sub-micron spatial resolution. In contrast, TXM reveals dentinal tubules in greater detail, allowing the observation of features such as miniscule lateral branches exhibiting complex 3D orientations. Figure 1B shows the 3D reconstruction of the distal ends of the dentinal tubules. We observe that most distal ends of the dentinal tubules with a 100-nm diameter are curved (white arrow in Fig. 1B) and end at the DEJ, and some dentinal tubules extend vertically through the DEJ, forming ESs. The diameters of the proximal end of the dentinal tubules increase and form elliptical shapes exhibiting tiny lateral branches. The 3D tomography results shown in Fig. 1C reveal structural defects (indicated by white arrows). These fragmented dentinal tubules might have been caused by external forces, diseases, or fossilization processes, providing essential information on the histopathological affections and preservation conditions.

**Figure 2:**
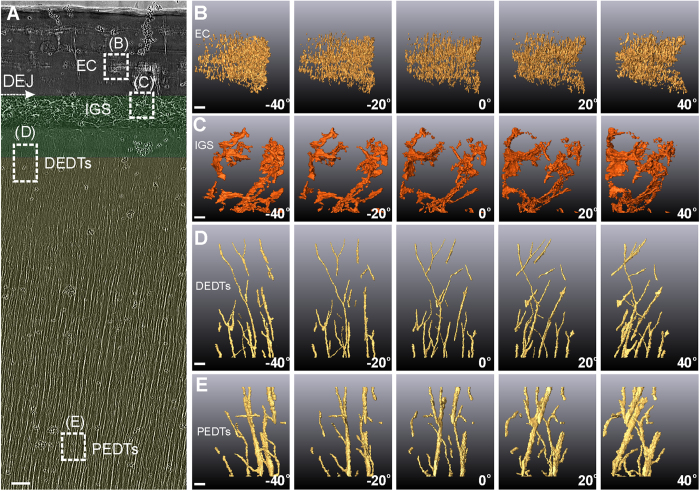
Ultrahigh-resolution internal microstructures of a *Tarbosaurus* tooth obtained via TXM. (**A**) 2D X-ray photograph of a sectioned *Tarbosaurus* tooth. Mantle dentin and dentin are colored translucent green and yellow, respectively. Enamel is uncolored. (Scale bar: 10 μm.) White-dashed rectangles indicate the 3D reconstruction regions shown in (**B**–**E**): (**B**) enamel crack (EC); (**C**) interglobular porous space (IGS) structures; (**D**) distal ends of the dentinal tubules (DEDTs); and (**E**) proximal ends of the dentinal tubules (PEDTs), which are approximately 190 μm away from the DEJ. (Scale bars: 1 μm.) (The small black dots in (**A**) are gold particles, which are used for system and image positioning).

The internal microstructures of the *Tarbosaurus* (Saurischia) tooth (Fig. 2) exhibit three marked differences in configuration as compared to the *Saurolophus* tooth: First, in *Tarbosaurus*, the enamel does not exhibit ESs, although miniscule parallel enamel cracks are apparent (Fig. 2B). Second, mantle dentin (MD) appears above the distal end of the dentinal tubules and below the DEJ (Fig. 2A). Some complex mesh-like crimped hollow microstructures can be observed in the MD. And most importantly, no dentinal tubules exhibit in this region. These interglobular porous spaces (IGS) inside the MD (see Fig. 2C and Supplementary Movie S3) may be formed by non-fully merged calcospheritic structures^25^. Similar complex porous structures can also be observed near the cementodentinal junction of human or some mammalian teeth, called Tome’s granular layer of dentin^26^,^27^,^28^. But that is only in the roots, where there is no enamel. This three-tissue compositional tooth (enamel–MD–bulk dentin) near the DEJ is the most significant difference from the two-tissue compositional (enamel–bulk dentin) teeth of *Saurolophus*. Third, the distal end of the dentinal tubules exhibit unique branching features (Fig. 2D). The diameter of these branching tubules eventually becomes so small that a gap seems to exist between the IGS and the distal end of the dentinal tubules. The region of scarce dentinal tubules is also a typical feature of MD. In both saurischian and ornithischian dinosaurs, however, the proximal end of the dentinal tubules exhibit similar lateral communication canals with a diameter of approximately half a micron extending from the main tubules (Fig. 2E).

**Figure 3:**
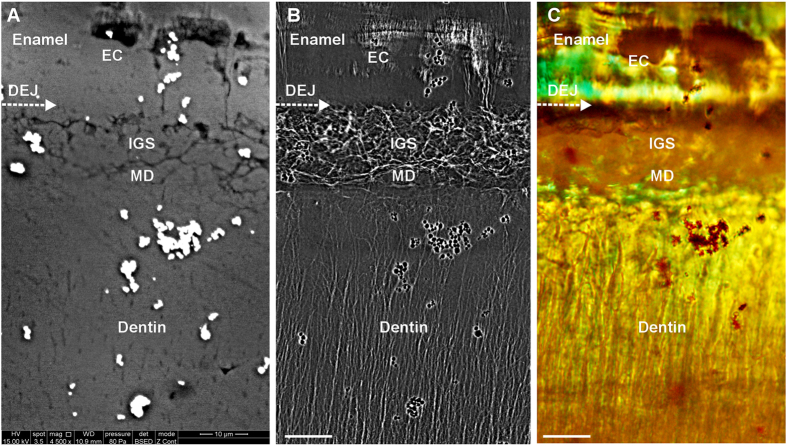
Comparison of SEM, TXM, and polarized light images of the internal structures of a *Tarbosaurus* tooth near the DEJ: (A) SEM back-scattering image, (B) TXM image, and (C) polarized light image captured using a 100 × objective. The aquamarine color in this figure represents enamel birefringence, and helps to identify the enamel region. (Scale bars: 10 μm.) (The bright dots in (**A**) and black dots in (**B**) and (**C**) are gold particles, which were used for image and system positioning.)

Figure 3 compares the SEM, TXM, and polarized light images of a *Tarbosaurus* tooth. The SEM image reveals only the parts of the IGS, enamel cracks, and dentinal tubules exposed to the specimen surface (Fig. 3A). By comparison, the TXM image displays in greater detail the fine structure embedded within the tooth (Fig. 3B). The polarized light images facilitate the identification of enamel based on the enamel birefringence effect, which displays a significant wavy pattern on enamel (see Fig. 3C, Supplementary Fig. S1A and Fig. S3A), but exhibits fewer fine structures within the tooth because of the spatial resolution, focal depth, and penetration limitations of this technique.

**Figure 4:**
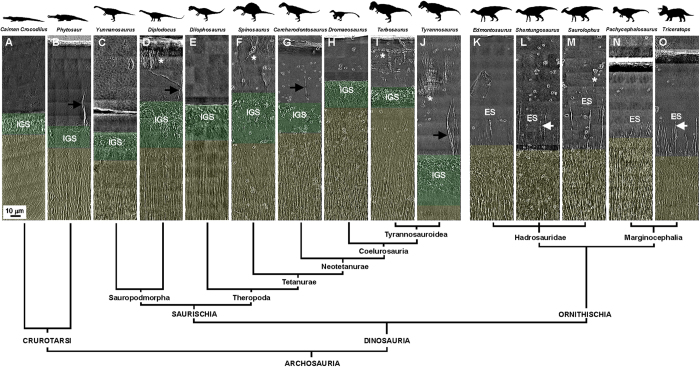
Internal tooth microstructures of various dinosaur genera within a comparative phylogenetic framework: (A) Extant *Caiman Crocodilus*, (B) *Phytosaur* (ROM 7981), (C) *Yunnanosaurus*, (D) *Diplodocus*, (E) *Dilophosaurus*, (F) *Spinosaurus*, (G) *Carcharodontosaurus*, (H) *Dromaeosaurus*, (I) *Tarbosaurus*, (J) *Tyrannosaurus*, (K) *Edmontosaurus*, (L) *Shantungosaurus*, (M) *Saurolophus*, (N) *Pachycephalosaurus*, and (O) *Triceratops*. Asterisks indicate enamel cracks. Black and white arrows indicate enamel tufts (ET) and the periodic features of a long enamel spindle (LES), respectively. Here, IGS: interglobular porous space structure; ES: enamel spindle; and ROM: Royal Ontario Museum. Mantle dentin and dentin are colored translucent green and yellow, respectively. Enamel is uncolored. (The small black dots in (**A**–**O**) are gold particles, which were used for system and image positioning.)

The organizational pattern differences in the internal tooth microstructures of *Saurolophus* and *Tarbosaurus* may suggest evolutionary diversity. To test this hypothesis, we examined other dinosaur teeth from Ornithischia and Saurischia to identify a possible taxonomic pattern among these dinosaurs, based on the prominent features of their teeth. The dinosaurs included eight saurischian and five ornithischian dinosaurs from the Early Jurassic to the Late Cretaceous Periods. Figure 4 shows high-resolution 2D X-ray images of transverse sections of the teeth, while the sectioning regions of the teeth are depicted in Fig. S2. Surprisingly, all five ornithischian dinosaur teeth exhibit long and straight ESs, extending from the underlying dentinal tubules through the DEJ. From a structural point of view, no MD was detected between the enamel and bulk dentin, because dentinal tubules must be absent or scarce in MD (Fig. 4K–O). However, all eight saurischian dinosaur teeth exhibited MD with IGS structures near the DEJ (Fig. 4C–J). Without exception, MD with IGS structures and ESs appeared in the saurischian and ornithischian teeth near the DEJ respectively in our random selection of thirteen dinosaurs.

## Discussion

Dietary preference often evolved in concert with dental modification^5^. However, our anatomical analysis suggests that dietary preference may not have influenced significantly the internal microstructures and their configurations, but mainly the morphologies of a dinosaur’s teeth, both in Saurischia and Ornithischia. For example, there are various dietary preferences in Saurischia, such as herbivorous Sauropodomorpha (Fig. 4C–D) and carnivorous Theropoda (Fig. 4E–J). The internal microstructures of their teeth near the DEJ exhibit similar three-tissue compositional patterns. In addition, although Ornithischia are all herbivores, the dental morphologies are quite different between taxa. For instance, the overall morphology of *Pachycephalosaurus* teeth is quite different from that of hadrosaurid teeth, but the internal microstructures of their teeth still exhibit a similar two-tissue organization pattern near the DEJ (Fig. 4K–O). This implies that the overall mechanical properties of dinosaur teeth may change through morphological modification to adapt to their preferred foods, but the fundamental tooth formation strategy did not change significantly.

Primitive reptilian teeth have been characterized as having two-tissue organization, including enamel and orthodentin. However, our observations show that the compositions of dinosaur teeth are much more complex than in primitive reptilian teeth, both in Saurischia and Ornithischia. For example, in Saurischia, MD is also a critical tissue in their teeth. Although the precise function of MD remains unclear, this hypocalcified tissue is thought likely to improve overall tooth elasticity. Some studies have shown that MD is generally softer than the above enamel and the underlying bulk dentin^29^,^30^. This additional cushioning soft layer may have functioned as a shock absorption system that helped reduce stress propagation from enamel to dentin and prevented cracks in the brittle enamel from extending into the dentin. In addition, IGS structures inside MD may also have contributed to the resilience of dinosaur teeth and provided stress shielding inside the teeth.

**Figure 5:**
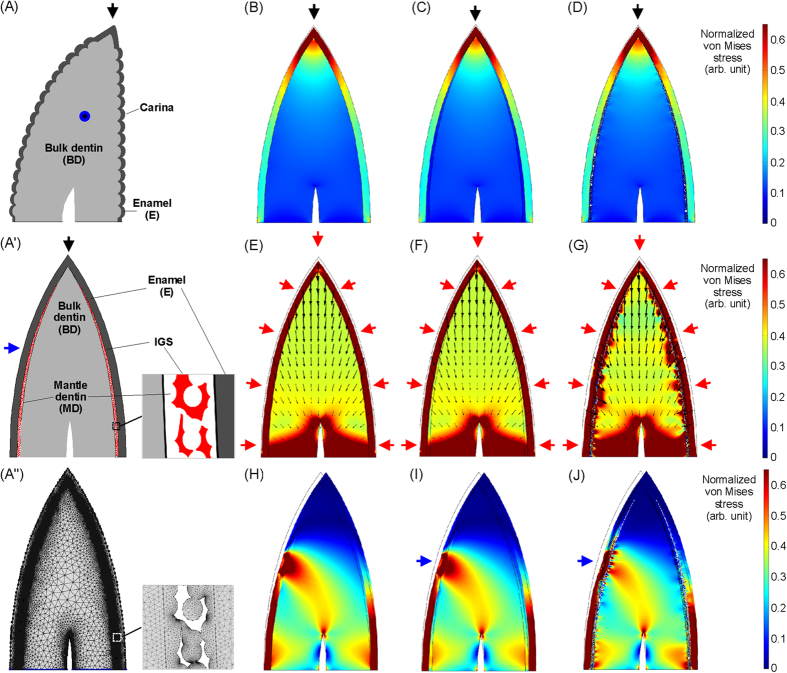
Two-dimensional (2D) multi-tissue mechanical simulations of a long and sharp saurischian tooth. (**A**) Lingual view of a saurischian tooth. (**A’**) Mesial or distal view of a saurischian tooth. (**A”**) Mesh structure of the tooth model generated by FEA software. This 2D geometric model is used in the simulations. Black, blue, and red arrows show the directions of applied external forces. (**B–D**) A force acts on the apex of a saurischian tooth, which consists of various dental compositions near the dentinoenamel junction (DEJ), such as E–BD (**B**), E–MD–BD (**C**), and E–MD–BD, with interglobular porous space (IGS) structures (**D**). (**E**–**G**) A net force acts normally on the whole enamel surface of a saurischian tooth, which includes various dental compositions near the DEJ, such as E–BD (**E**), E–MD–BD (**F**), and E–MD–BD with IGS structures (**G**). Small black arrows inside the tooth show the distribution of the displacement field inside it when an external loading is applied to it. The pointing direction and length of an arrow are the direction and relative magnitude of the displacement, respectively. (**H**–**J**) A force acts on the lingual or labial surface of a saurischian tooth, which is consists of various dental compositions near the DEJ, such as E–BD (**H**), E–MD–BD (**I**), and E–MD–BD, with IGS structures (**J**). Here, E: enamel; BD: bulk dentin; and MD: mantle dentin.

To further examine this hypothesis, we used finite-element analysis (FEA, Comsol Multiphysics software) to explore the mechanical functions of MD and IGS structures inside a typical saurischian tooth. Figure 5 shows two-dimensional mechanical simulations of a long and sharp saurischian tooth with multi-tissue composition that is under external stress. The IGS structures are simulated by obtaining the interspaces between randomly distributed circles. The distribution of IGS structures inside the MD in the simulation model (Fig. 5A’) is based on the observation in Supplementary Fig. S3. The thickness of the MD gradually decreased closer to the tooth apex, as shown in the *Dromaeosaurus* teeth (Supplementary Fig. S3A). This indicates that the IGS structures as well as the MD at the apex region are almost absent. Similar result can be observed inside an extant *Caiman Crocodilus* tooth as shown in Supplementary Fig. S3B–D as well. Because the mechanical properties of fossil teeth may be changed during fossilization process, the elastic modulus parameters of human enamel, MD, and bulk dentin are used in the simulation, based on refs 31,32. They are 63.6, 19.7, and 26.5 GPa, respectively. The Poisson’s ratios of human dentin and enamel used in the simulations are 0.31 and 0.33^33^. Figure 5A” shows the mesh structure generated by the FEA software, which was used in the simulations.

Three kinds of mechanical situations were simulated. In the first situation, stress acts on the tooth apex, simulating a saurischian dinosaur catching a prey using its tooth apex. We compared three kinds of dental configurations near the DEJ in the simulations, including two-tissue composition, three-tissue composition, and three-tissue composition with IGS structures inside the MD. Because both MD and IGS structures are not present at the apex region, the simulation results show that, when the tooth apex just touches the prey’s tissue surface, the stress distribution at the apex region is almost unchanged among these three kinds of configurations (Fig. 5B–D). It is worth noting that the stress is highly concentrated at the enamel apex, owing to the sharp geometry, which may help teeth to strike or penetrate prey tissues much more easily. It makes sense that the tissues at the apex region need to offer sufficient stabilization and hardness to perform deliberate penetration and, therefore, soft tissues such as MD do not provide any apparent benefit in this region. The other region that had a mechanical function in saurischian teeth is the carina on the mesial and distal sides^34^,^35^. The carinae provide a cutting function and, therefore, no MD was present between the enamel and bulk dentin in these regions is reasonable^35^.

In the second situation, when a saurischian dinosaur penetrates their prey using long and sharp teeth, not only the penetration force but also the extrusion forces will apply on the tooth surfaces, exerted by the punctured prey tissues around the teeth. In order to understand the mechanical roles of MD and IGS inside a saurischian tooth in this puncturing process, we apply a net force normal to the external enamel surface to simulate the combination force acted on the tooth (Fig. 5E–G). The simulation results show that the direction of the resulting net force inside a tooth is almost apply to the tooth root, because the symmetric lateral forces are balanced inside the tooth. No significant differences in the tooth-internal stress distributions were visible between these three configurations. However, the resulting displacement fields near the enamel shows that the configuration characterized by a three-tissue composition with IGS structures inside the MD provides a larger spatial buffer than the other two configurations when the same loading is applied on the external enamel surface. It is possible that brittle enamel against surrounding punctured prey tissues may be easily ruptured by the tight extrusion force when a tooth strenuously penetrate into a hard tissue without any underlying cushioning protection mechanism. Another advantage of this large elastic buffer inside saurischian teeth may be that it helps the teeth to extract from hard tissues more easily.

In the third situation, stress acts on the sides of the teeth. For example, dinosaurs likely shook the prey to subdue or kill it as extant crocodiles do. Under these conditions, force mainly acts on the lingual or labial surfaces of a tooth: this asymmetric lateral or bending force is one of the dominant destructive forces responsible for tooth fracture, especially for long teeth with a high aspect ratio. The simulation results show that the three-tissue compositional tooth has better stress shielding ability than the two-tissue compositional tooth (Fig. 5H,I). Similar multi-tissue configurations with different elasticities have been shown to improve material toughness, such as in shells, nacre, and some bio-inspired materials^36^,^37^. It is important to note that the three-tissue compositional tooth with IGS structures inside the MD provides better stress shielding ability than the other configurations (Fig. 5H–J). The simulation results show that the randomly distributed IGS structures can redistribute stress on the enamel and shield the stress, which can prevent intense stress from propagating to the weaker bulk dentin and causing tooth fracture. Thus, soft MD with IGS structures within long and sharp saurischian teeth provides protection buffer and an increased fractural threshold as a tooth being hit by destructive lateral forces.

**Figure 6:**
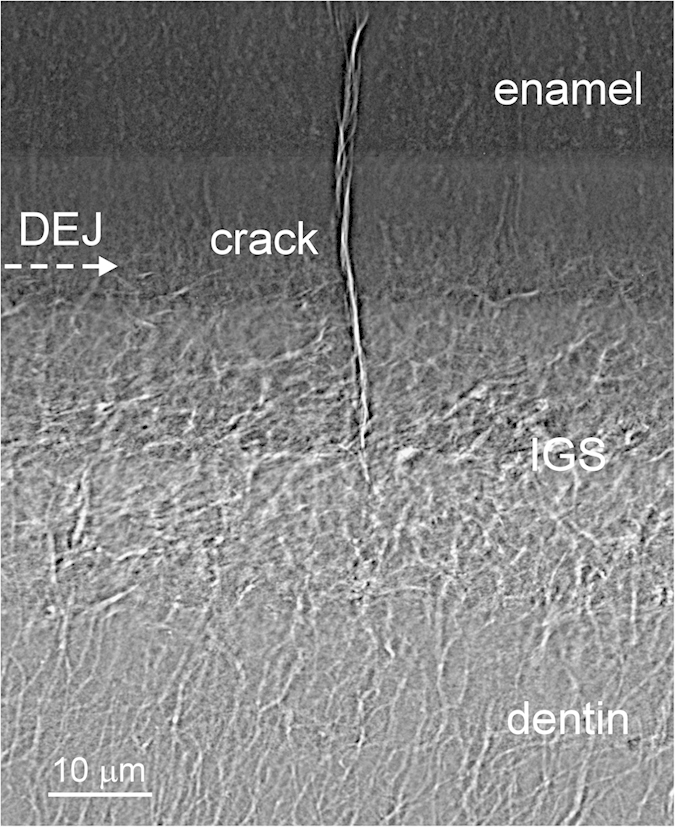
An enamel crack was arrested at interglobular porous space (IGS) region analyzed by using TXM. A deep enamel crack observed in an extant *Caiman Crocodilus* tooth was arrested inside the IGS region rather than at the DEJ boundary. Because, the IGS is a kind of randomly distributed porous structure that may help redirect crack propagation and release the stress. The results also demonstrated that the high-resolution TXM image can help us identify the crack-arrested site or crack trajectory in teeth much more clearly.

Some studies have proposed that enamel cracks produced by external forces can be arrested at the DEJ or MD, owing to the elastic mismatch between enamel and dentin^31^. To eliminate complex crack formation possibilities inside fossil teeth, we used extant crocodilian tooth as a model, since this reptile exhibited similar tissue composition near DEJ to that of saurischian dinosaurs. Our observations revealed that deepest enamel cracks observed inside the extant crocodilian tooth were arrested at the IGS region inside the MD (Fig. 6). The IGS structures may play a crucial role in arresting cracks inside teeth as well. We propose that these randomly distributed porous structures can redirect crack propagation and release stress, which prevents cracks from penetrating into bulk dentin and causing tooth fracture.

**Figure 7:**
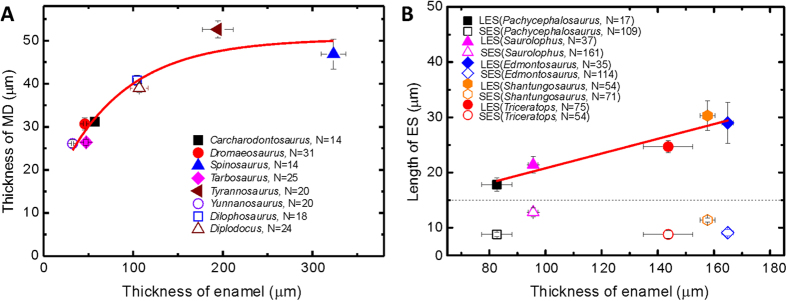
Quantitative analysis of the thicknesses of mantle dentin (MD) and enamel, as well as the lengths of long and short enamel spindles (LES and SES). (**A**) Thickness relationships between MD and enamel of saurischian dinosaurs. Each data point was collected from randomly selected regions on each of the eight saurischian dinosaur teeth analyzed in this study. In this diagram, the thickness of MD is significantly correlated with the thickness of enamel as a *y* = a × (1 − exp(−*x/*t)) + b function. Where *y* is MD thickness, *x* is the enamel thickness, and a + b is the maximum MD thickness, which is about 50 μm. (**B**) The relationship between enamel spindle length and enamel thickness in ornithischian dinosaurs. Each data point was collected from three randomly selected regions on each of the five ornithischian dinosaur teeth analyzed in this study. The average lengths of the SESs of the five ornithischian dinosaurs were approximately 10 μm, and were not associated with the genera or enamel thicknesses. In contrast, the statistical lengths of the LESs ranged from 18 to 30 μm, linearly depending on the enamel thicknesses, which can be fitted by a linear function expressed as *y* = c + d × *x*. Where *y* is the length of LES, *x* is the enamel thickness, c is the intercept (7.46 μm), and d is the slope (0.14) of this function. Error bars represent 95% confidence intervals determined according to the Student’s *t*-test distribution.

The thickness of MD varies according to genus, and appears to be associated with enamel thickness in an exponential relationship [~a × (1 − exp(−*x/*t)) + b], where *x* is the enamel thickness, t is a constant about 74.4 μm, and a + b is the maximum thickness of MD (Fig. 7A). One possible interpretation is that, compared to thin enamel, thick enamel requires a stronger elastic shock absorption system to reduce stress propagation. However, the maximum thickness of MD in saurischian teeth is limited to ~50 μm, even if the above enamel thickness increases further. This indicates that the thickness of MD may be limited by its formative mechanism.

Other dental protection mechanism formed by internal microstructures has also been observed inside some saurischian teeth. Chai^38^ demonstrated that a crack-like internal microstructure of enamel tufts—a type of hypomineralized and organic-matrix-filled defect inside enamel—inside human teeth may play an important role in damage resistance and crack shielding. In our observations, some saurischian teeth, such as *Diplodocus*, *Carcharodontosaurus*, and *Tyrannosaurus*, exhibited enamel tufts near the DEJ as well (black arrows in Fig. 4D,G,J).

The complex dental composition in hadrosaurid ornithischians forms a file-like grinding surface for differential grinding on hard and tough plants^2^. Our observations show that little or no MD was present between enamel and bulk dentin in these ornithischian teeth (see Fig. 4K–O and Supplementary Fig. S4A). One possible reason is that the entirety of the enamel in ornithischian teeth was used for food processing, hence the stabilization and hardness of enamel needed to be maintained. However, MD with IGS structures can also be observed in ornithischian teeth and is present only between the cementum and bulk dentin (Supplementary Fig. S4B). These structures are generally called Tome’s granular layers. They are similar to the grinding teeth of most mammals and have a protective function for the entire tooth. From the perspective of dental function, the three-tissue composition near the DEJ may be much more suitable for long and sharp saurischian hunting teeth than the two-tissue composition, and provides the necessary protection when the teeth are performing penetration. In contrast, the loss of soft MD between enamel and bulk dentin in ornithischian teeth may be better for the purpose of grinding or slicing foods, for example, by influencing the formation of a file-like grinding surface^2^,^39^. The results also imply that the presence of MD beneath the DEJ is one of the most important differences between saurischian and ornithischian teeth.

In order to determine the primitive compositional condition of teeth near the DEJ in dinosaurs, we examined the internal microstructures of teeth from the outgroup taxa in Crurotarsi, including extant crocodile (Fig. 4A) and extinct phytosaur (Fig. 4B). Both teeth reveal similar MD with IGS structures near the DEJ, implying that the three-tissue composition near the DEJ that appears to be the primitive condition. Thus, the two-tissue composition near the DEJ of ornithischian teeth is the result of subsequent evolution, possibly related to the feeding mechanism for these herbivorous dinosaurs.

In addition to the three-tissue and two-tissue compositional differences, other internal features of dinosaur teeth may also serve as the basis for a new method for dinosaur taxonomic identification and has future potential for phylogenetic analyses. Although the enamel of both the *Tarbosaurus* and *Tyrannosaurus* teeth exhibit minute enamel cracks (asterisks in Fig. 4I,J), the enamel characteristics are distinct. In *Tarbosaurus* enamel, the cracks are short and parallel, and exhibit two formed layers. In contrast, the cracks in *Tyrannosaurus* enamel exhibit a bamboo-broom-like pattern. Enamel tufts are another prominent feature in some saurischian teeth as well (Fig. 4D,G,J). Furthermore, the ESs inside ornithischian teeth exhibited some differences. For example, the 3D periodic feature of ESs is observed in *Shantungosaurus* and *Triceratops* teeth (white arrows in Fig. 4L,O, and Supplementary Fig. S5), but did not appear in the other three ornithischian genera (Figs 1B and 4K,MN). Moreover, the lengths of the LESs appeared to significantly correlate with the thickness of the enamel (Fig. 7B), but the lengths of the SESs of various ornithischian dinosaurs were highly similar. Table 1 compares the significant features of the teeth of various dinosaur species. Tooth-based characters related to the internal crown ultramicrostructure of the analyzed teeth are scored in Supplementary Table S1: we provide this information in anticipation of detailed cladistic analysis in the future. We suggest that combining high-resolution dental information near the DEJ with traditional morphological descriptions will provide another and more convincing source of distinguishable evidence for use in dinosaur identification and classification.

Using TXM, we demonstrated that the detailed, fine 3D internal microstructures of dinosaur teeth can be clearly identified with ultrahigh spatial resolution. This method facilitates an increased understanding of evolutionary processes associated with dinosaur teeth, and now observable within the teeth. Our results indicate that there is valuable anatomical, functional, and phylogenetic information within dental microstructure, and shows that the methodology also represents a new way of identifying and classifying dinosaur taxa. Our results also aid in understanding how the structural configuration and mechanical properties of dinosaur teeth may be related to their feeding behaviors. We believe that these results will complement existing paleontological analyses and provide new insights into the micro-/nano-scale paleontological research of ancient organisms.

## Methods

The fossils were sliced to a thickness of 50–100 μm using a Leica SP1600 Saw Microtome, and were then carefully hand-polished to a thickness of 20–30 μm. The processes of physical dissection and surface polishing do not affect the structures embedded inside fossils. This method also minimizes the breakage of fossils because it requires only a small portion of the specimen for observation. No other complex preparation processes were required for the observations, and as such, the original internal structures of the specimens were not destroyed during the sample preparation. Polarized light images were captured using an Olympus BX51 with a 50x objective. An FEI Quanta 200 Scanning Electron Microscope was also used to capture the SEM images of the fossil specimens. The SEM was operated under a vacuum of approximately 100 Pa, and the specimens were uncoated. The TXM facility and beamline BL01B at the Taiwan Light Source (TLS) in Hsinchu, Taiwan provided 2D imaging and 3D tomography at a spatial resolution of 30–60 nm. A superconducting wavelength shifter source provided a photon flux of 4 × 10^11^ photons s^−1^ (0.1% bw)^−1^ in the energy range of 5–20 keV. A double crystal monochromator utilizing a pair of Ge(111) crystals selected X-rays with an energy of 8–11 keV. The image of the specimen was magnified using a Fresnel zone plate, which served as an objective lens to magnify the images 44 × in the first-order diffraction mode. In conjunction with 20 × downstream optical magnification, this microscope provided a total magnification of 880 × for the first-order mode. In this mode of the zone plate, the field of view of the image is 15 × 15 μm^2^. A millimeter-scale field of view of the specimen can also be produced by stitching images from a series of observation positions. A higher spatial resolution, e.g. 30-nm, can be achieved using the third-order diffraction mode of the zone plate. After acquiring a series of 2D images with the sample rotated stepwise, 3D tomography data sets were reconstructed by applying a filtered back-projection algorithm based on 151 sequential image frames taken with the azimuth angle rotating from −75° to +75°. The final 3D tomography structures were generated using Arima 3D software to improve visualization.

## Additional Information

**How to cite this article**: Wang, C.-C. *et al.* Evolution and Function of Dinosaur Teeth at Ultramicrostructural Level Revealed Using Synchrotron Transmission X-ray Microscopy. *Sci. Rep.* **5**, 15202; doi: 10.1038/srep15202 (2015).

## Supplementary Material

Supplementary Information

Supplementary Movie S1

Supplementary Movie S2

Supplementary Movie S3

## Figures and Tables

**Table 1:** List of significant features of various dinosaur teeth.

Clades (museum number)	DP	ES	MD	PES	EC	ETs	ETh (μm)	TMD (μm)	LLES (μm)
*Yunnanosaurus* (WDV-09-11)	H	—	√	—	—	—	32.3 ± 1.2	26.1 ± 0.9	—
*Diplodocus* (NTM I06245)	H	—	√	—	√	√	106.8 ± 10.0	39.0 ± 1.1	—
*Dilophosaurus* (WDV-04-09)	C	—	√	—	—	√	104.2 ± 4.0	40.8 ± 1.2	—
*Spinosaurus* (NTM I06249)	C	—	—	—	√	√	323.6 ± 13.7	46.9 ± 3.5	—
*Carcharodontosaurus* (NTM I06247)	C	—	√	—	—	√	57.1 ± 2.4	31.2 ± 1.0	—
*Dromaeosaurus* (NTM I06250)	C	—	—	—	—	√	46.0 ± 1.7	30.7 ± 1.4	—
*Tarbosaurus* (NTM I06243)	C	—	—	—	√	√	47.4 ± 2.4	26.4 ± 0.7	—
*Tyrannosaurus* (NTM I06246)	C	—	√	—	√	√	194.4 ± 17.2	52.6 ± 2.0	—
*Edmontosaurus* (NTM I06248)	H	√	—	—	—	—	164.9 ± 2.0	—	29 ± 3.7
*Shantungosaurus* (GMV 1780-1)	H	√	—	√	—	—	157.7 ± 2.7	—	30.3 ± 2.7
*Saurolophus* (NTM I06242)	H	√	—	—	√	—	95.6 ± 1.7	—	21.4 ± 1.5
*Pachycephalosaurus* (NTM I06244)	H	√	—	—	—	—	82.7 ± 5.4	—	17.8 ± 1.2
*Triceratops* (ROM 67669)	H	√	—	√	—	—	143 ± 14.7	—	24.7 ± 1.1

A dinosaur genus may be identified using the features in the list. For example, although *Edmontosaurus* and *Pachycephalosaurus* teeth exhibit similar internal features, the enamel thicknesses (ETh) and lengths of LESs (LLES) can still be identified. The errors of the thickness of mantle dentin (TMD) and LLES are 95% confidence intervals determined according to Student’s *t-*test distribution. Here, DP: dietary preference; C: carnivorous; H: herbivorous; ES: enamel spindle; MD: mantle dentin near dentinoenamel junction; PES: periodic enamel spindle; EC: enamel crack; ET: enamel tuft; WDV: World Dinosaur Valley; NTM: National Taiwan Museum; GMV: Geological Museum of China, Vertebrate; and ROM: Royal Ontario Museum.

## References

[b1] Williams V. S., Barrett P. M. & Purnell M. A. Quantitative analysis of dental microwear in hadrosaurid dinosaurs, and the implications for hypotheses of jaw mechanics and feeding. Proc. Natl. Acad. Sci. USA 106, 11194–11199 (2009). 19564603 10.1073/pnas.0812631106 PMC2708679

[b2] Erickson G. M. *et al.* Complex dental structure and wear biomechanics in hadrosaurid dinosaurs. Science 338, 98–101 (2012). 23042891 10.1126/science.1224495

[b3] Low W. A. & Cowan I. M. Age determination of deer by annular structures of dental cementum. J. Wildlife Manage. 27, 466–471 (1963).

[b4] Dellabianca N. A. *et al.* Influence of climate oscillations on dentinal deposition in teeth of Commerson’s dolphin. Global Change Biol. 18, 2477–2486 (2012).

[b5] Mihlbachler M. C., Rivals F., Solounias N. & Semprebon G. M. Dietary change and evolution of horses in north America. Science 331, 1178–1181 (2011). 21385712 10.1126/science.1196166

[b6] Teaford M. F., Smith M. M. & Ferguson M. W. J. Development, function and evolution of teeth. (Cambrige University Press, 2000).

[b7] Currie P. J., Hurum J. H. & Sabath K. Skull structure and evolution in tyrannosaurid dinosaurs. Acta Palaeontol. Pol. 48, 227–234 (2003).

[b8] Samman T., Powell G. L., Currie P. J. & Hills L. V. Morphometry of the teeth of western north American tyrannosaurids and its applicability to quantitative classification. Acta Palaeontol. Pol. 50, 757–776 (2005).

[b9] Hendrickx C. & Mateus O. Abelisauridae (Dinosauria: Theropoda) from the Late Jurassic of Portugal and dentition-based phylogeny as a contribution for the identification of isolated theropod teeth. Zootaxa 3759, 1–74 (2014). 24869965 10.11646/zootaxa.3759.1.1

[b10] Hendrickx C., Mateus O. & Araújo R. The dentition of megalosaurid theropods. Acta Palaeontologica Polonica 60, 627–642 (2015).

[b11] Hwang S. H. Phylogenetic patterns of enamel microstructures in dinosaur teeth. J. Morphol. 266, 208–240 (2005). 16163689 10.1002/jmor.10372

[b12] Hwang S. H. The utility of tooth enamel microstructure in identifying isolated dinosaur teeth. Lethaia 43, 307–322 (2010).

[b13] Hwang S. H. The evolution of dinosaur tooth enamel microstructure. Biol. Rev. 86, 183–216 (2011). 20518758 10.1111/j.1469-185X.2010.00142.x

[b14] Sutton M. D. Tomographic techniques for the study of exceptionally preserved fossils. P. Roy. Soc. B-Biol. Sci. 275, 1587–1593 (2008). 10.1098/rspb.2008.0263 PMC2394564 18426749

[b15] Donoghue P. C. J. *et al.* Synchrotron X-ray tomographic microscopy of fossil embryos. Nature 442, 680–683 (2006). 16900198 10.1038/nature04890

[b16] Earl J. S. *et al.* Characterization of dentine structure in three dimensions using FIB-SEM. J. Microsc.-Oxford 240, 1–5 (2010). 10.1111/j.1365-2818.2010.03396.x 21050207

[b17] Yin G. C. *et al.* Energy-tunable transmission x-ray microscope for differential contrast imaging with near 60 nm resolution tomography. Appl. Phys. Lett. 88, 241115 (2006).

[b18] Song Y. F. *et al.* X-ray beamlines for structural studies at the NSRRC superconducting wavelength shifter. J. Synchrotron Radiat. 14, 320–325 (2007). 17587656 10.1107/S0909049507021516

[b19] Yin G. C. *et al.* 30 nm resolution x-ray imaging at 8 keV using third order diffraction of a zone plate lens objective in a transmission microscope. Appl. Phys. Lett. 89, 221122 (2006).

[b20] Avery J. K., Steele P. F. & Avery N. Oral Development and Histology. 3rd edn, 162–163 (Thieme, 2002).

[b21] Parkinson C. R. & Sasov A. High-resolution non-destructive 3D interrogation of dentin using X-ray nanotomography. Dent. Mater. 24, 773–777 (2008). 17964644 10.1016/j.dental.2007.09.003

[b22] Zaslansky P., Zabler S. & Fratzl P. 3D variations in human crown dentin tubule orientation: A phase-contrast microtomography study. Dent. Mater. 26, e1–e10 (2010). 19879641 10.1016/j.dental.2009.09.007

[b23] Liu M.-H., Chan C.-H., Ling J.-H. & Wang C. R. C. Filling in dentinal tubules. Nanotechnology 18, 475104 (2007).

[b24] Brannstrom M. & Brhannstrhom M. Detin and Pulp in Restorative Dentistry. (Wolfe Medical Publications, 1982).

[b25] Goldberg M., Kulkarni A. B., Young M. & Boskey A. Dentin: Structure, Composition and Mineralization: The role of dentin ECM in dentin formation and mineralization. Front. Biosci. 3, 711–735 (2012). 10.2741/e281 PMC3360947 21196346

[b26] Kagayama M., Sasano Y. & Tsuchiya M. Confocal microscopy of Tomes’ granular layer in dog premolar teeth. Anat. Embryol. 201, 131–137 (2000). 10672365 10.1007/pl00008233

[b27] Tsuchiya M., Sasano Y., Kagayama M. & watanabe M. Characterization of Interglobular Dentin and Tomes’ Granular Layer in Dog Dentin Using Electron Probe Microanalysis in Comparison with Predentin. Calcif. Tissue Int. 68, 172–178 (2001). 11351501 10.1007/s002230001208

[b28] Cate A. R. T. An analysis of Tome’s granular layer. Anat. Rec. 172, 137–147 (1972). 4111019 10.1002/ar.1091720202

[b29] Zaslansky P., Friesem A. A. & Weiner S. Structure and mechanical properties of the soft zone separating bulk dentin and enamel in crowns of human teeth: Insight into tooth function. Journal of Structural Biology 153, 188–199 (2006). 16414277 10.1016/j.jsb.2005.10.010

[b30] Wang R. Z. & Weiner S. Strain-structure relations in human teeth using Moir’e fringes. Journal of Biomechanics 31, 135–141 (1998). 9593206 10.1016/s0021-9290(97)00131-0

[b31] Bechtle S. *et al.* Crack arrest within teeth at the dentinoenamel junction caused by elastic modulus mismatch. Biomaterials 31, 4238–4247 (2010). 20167362 10.1016/j.biomaterials.2010.01.127

[b32] Jr. G. W. M., Balooch M., Gallagher R. R., Gansky S. A. & Marshall S. J. Mechanical properties of the dentinoenamel junction: AFM studies of nanohardness, elastic modulus, and fracture. Journal of Biomedical Material Research 54, 87–95 (2001). 10.1002/1097-4636(200101)54:1<87::aid-jbm10>3.0.co;2-z 11077406

[b33] Milewski G. Numerical and experimental analysis of effort of human tooth hard tissues in terms of proper occlusal loadings. Acta of Bioengineering and Biomechanics 7, 47–59 (2005).

[b34] Brink K. S. & Reisz R. R. Hidden dental diversity in the oldest terrestrial apex predator Dimetrodon. Nature Communications 5, 3269 (2014). 10.1038/ncomms4269 24509889

[b35] Brink K. S. *et al.* Developmental and evolutionary novelty in the serrated teeth of theropod dinosaurs. Scientific Reports 5, 12338 (2015). 26216577 10.1038/srep12338 PMC4648475

[b36] Suresh S. Graded Materials for Resistance to Contact Deformation and Damage. Science 292, 2447–2451 (2001). 11431558 10.1126/science.1059716

[b37] Munch E. *et al.* Tough, Bio-Inspired Hybrid Materials. Science 322, 1516–1520 (2008). 19056979 10.1126/science.1164865

[b38] Chai H., Lee J. J.-W., Constantino P. J., Lucas P. W. & Lawn B. R. Remarkable Resilience of Teeth. Proc. Natl. Acad. Sci. USA 106, 7289–7293 (2009). 19365079 10.1073/pnas.0902466106 PMC2678632

[b39] Erickson G. M. *et al.* Wear biomechanics in the slicing dentition of the giant horned dinosaur Triceratops. Science Advances 1, 1–7 (2015). 10.1126/sciadv.1500055 PMC4640618 26601198

