# Supplementary material for: Evolution and Function of Dinosaur Teeth at Ultramicrostructural Level Revealed Using Synchrotron Transmission X-ray Microscopy
Source: Sci Rep. 2015 Oct 29;5:15202. doi: 10.1038/srep15202 (PMC4625602; doi:10.1038/srep15202)
Supplement: Supplementary Information [file srep15202-s1.pdf]

Supplementary Information

Evolution and Function of Dinosaur Teeth at Ultramicrostructural Level Revealed Using Synchrotron Transmission X-ray Microscopy

Chun-Chieh Wang[1*], Yen-Fang Song[1*], Sheng-Rong Song[2], Qiang Ji[3], Cheng-Cheng Chiang[1], Qingjin Meng[4], Haibing Li[3], Kiko Hsiao[5], Yi-Chia Lu[2], Bor-Yuan Shew[1], Timothy Huang[6,8], Robert R. Reisz[7,8*]

[1]National Synchrotron Radiation Research Center, 30076 Hsinchu, Taiwan.
[2] Department of Geosciences, National Taiwan University, 10617 Taipei, Taiwan.
[3] Institute of Geology, Chinese Academy of Geological Sciences, 100037 Beijing, China.
[4] Beijing Museum of Natural History, 100050 Beijing, China.
[5] Department of Geology, Chinese Culture University, 11114 Taipei, Taiwan
[6] Institute of Genomics and Bioinformatics, National Chung Hsing University, 402 Taichung, Taiwan..
[7] Department of Biology, University of Toronto Mississauga, Mississauga, Ontario L5L 1C6, Canada.
[8]Department of Optics and Photonics, National Central University, 32001 Tauyuan, Taiwan.

CORRESPONDING AUTHORS

Chun-Chieh Wang (E-mail: wang.jay@nsrrc.org.tw)
Yen-Fang Song (E-mail: song@nsrrc.org.tw)
Robert R. Reisz (E-mail: robert.reisz@utoronto.ca)

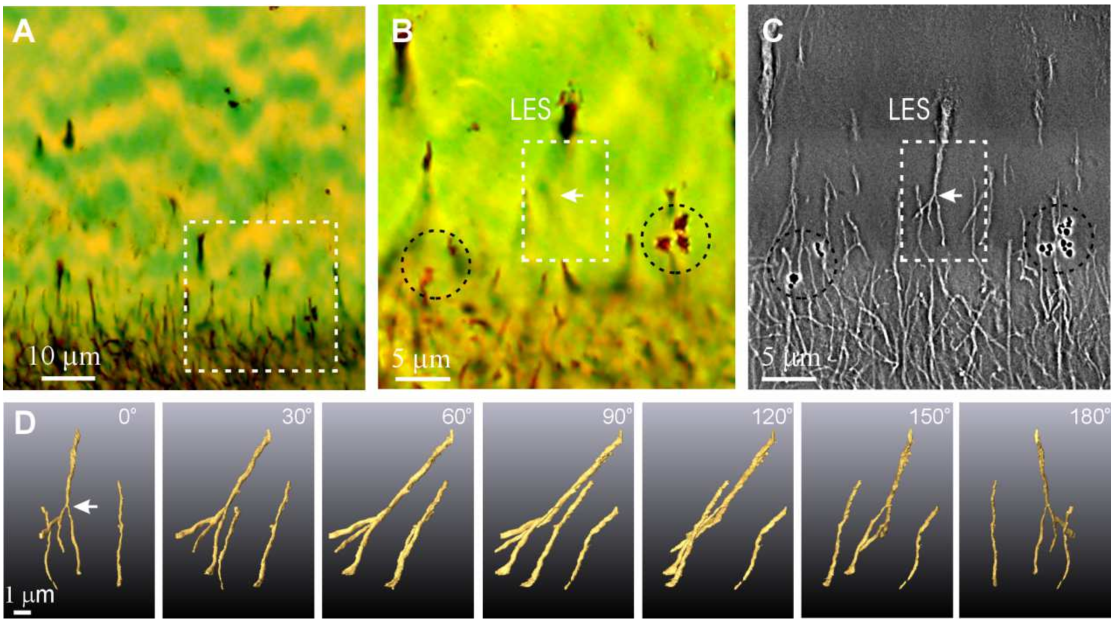

Figure S1. Structure of a long enamel spindle (LES) near the dentinoenamel junction (DEJ) in an *Edmontosaurus* tooth. (**A**) Polarized light image captured using a 20× objective. The wavy signal shows the region of the enamel. (**B**) Polarized light image captured using a 50× objective from the dashed-rectangular region in (**A**). (**C**) Ultrahigh-resolution 2D TXM image captured at the same region of (**B**). (**C**) Ultrahigh-resolution 3D tomography inside the dashed rectangular regions in (**A**) and (**B**). The white arrows indicate the combination sites of three dentinal tubule extensions becoming an LES.

Figure S1 shows the typical structure of a long enamel spindle (LES) near the dentinoenamel junction (DEJ) in ornithischian dinosaur teeth. Figure S1A and S1C show the corresponding conventional transmission polarized light microscopy and ultrahigh-resolution synchrotron transmission X-ray microscopy (TXM) images, respectively, of the internal structures of an *Edmontosaurus* tooth. The TXM image clearly shows the fine structures in full within the tooth. The polarized light image is much more blurry, and reveals less structural information than the TXM image. This blur is caused by the diffraction limit, small focus depth, and penetration depth of conventional optical imaging systems. Figure S1D shows the corresponding ultrahigh-resolution 3D structural reconstruction of the dashed rectangular region in Figs. S1B and S1C. This figure clearly shows three small dentinal tubules (DT) extended through the DEJ and combined to become an LES (see Supplementary Movie S2). The binding positions are indicated by white arrows in Fig. S1B–D, and the positioning of gold nanoparticles is marked by dashed black circles in Fig. S1A–C.

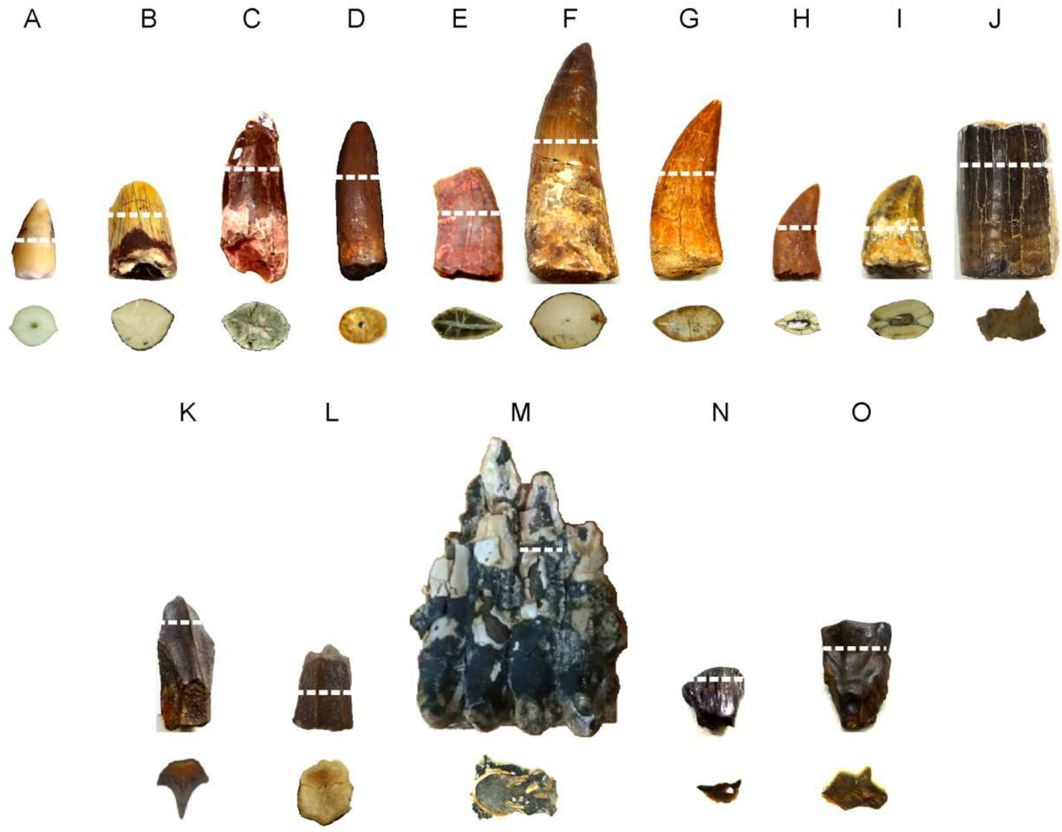

Figure S2. Transverse sections of teeth used in Fig. 4. White-dashed line indicates the sectioning plane position of each tooth. (**A**) Extant *Caiman Crocodilus* (intact tooth), (**B**) *Phytosaur* (fragment), (**C**) *Yunnanosaurus* (intact tooth), (**D**) *Diplodocus* (intact tooth), (**E**) *Dilophosaurus* (fragment), (**F**) *Spinosaurus* (intact tooth), (**G**) *Carcharodontosaurus* (intact tooth), (**H**) *Dromaeosaurus* (intact tooth), (**I**) *Tarbosaurus* (intact tooth), (**J**) *Tyrannosaurus* (fragment), (**K**) *Edmontosaurus* (intact tooth), (**L**) *Shantungosaurus* (fragment), (**M**) *Saurolophus* (battery), (**N**) *Pachycephalosaurus* (intact tooth), and (**O**) *Triceratops* (intact tooth).

Figure S2 shows the morphologies and thin sections of the teeth analyzed in this work. Some of the specimens are only tooth fragments, and not intact teeth (Figs. S2B, E, J, and L), or otherwise a fragment cut from the dental battery (Fig. S2M). The fact that the new taxon identification methodology works accurately even on non- intact teeth demonstrates its analytic advantages and potential.

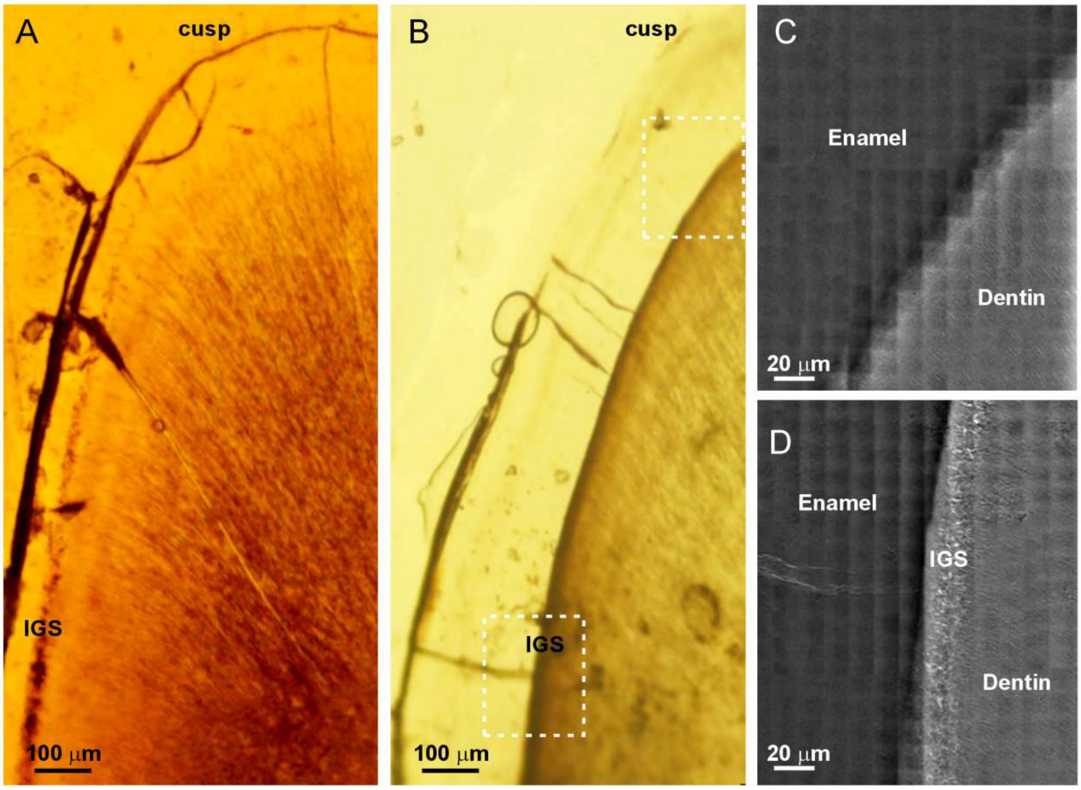

Figure S3. Distribution of interglobular porous space (IGS) structures in *Dromaeosaurus* and extant *Caiman Crocodilus* teeth. Transmission optical images of (**A**) a *Dromaeosaurus* tooth and (**B**) an extant *Caiman Crocodilus* tooth, captured near the cusp. High-resolution TXM images captured from (**C**) the upper dashed-rectangular region and (**D**) the lower dashed-rectangular region of (**B**).

Figure S3 shows the distribution of interglobular porous space (IGS) structures in *Dromaeosaurus* (Fig. S3A) and extant *Caiman Crocodilus* (Figs. S3B–D) teeth. We can see that the IGS structures become almost absent near the tooth cusp, both in *Dromaeosaurus* and extant *Caiman Crocodilus* teeth (Figs. S3A and C). The results also demonstrate that TXM images can help us identify the IGS distribution in teeth much more clearly than conventional optical microscopy (Figs. S3B–D).

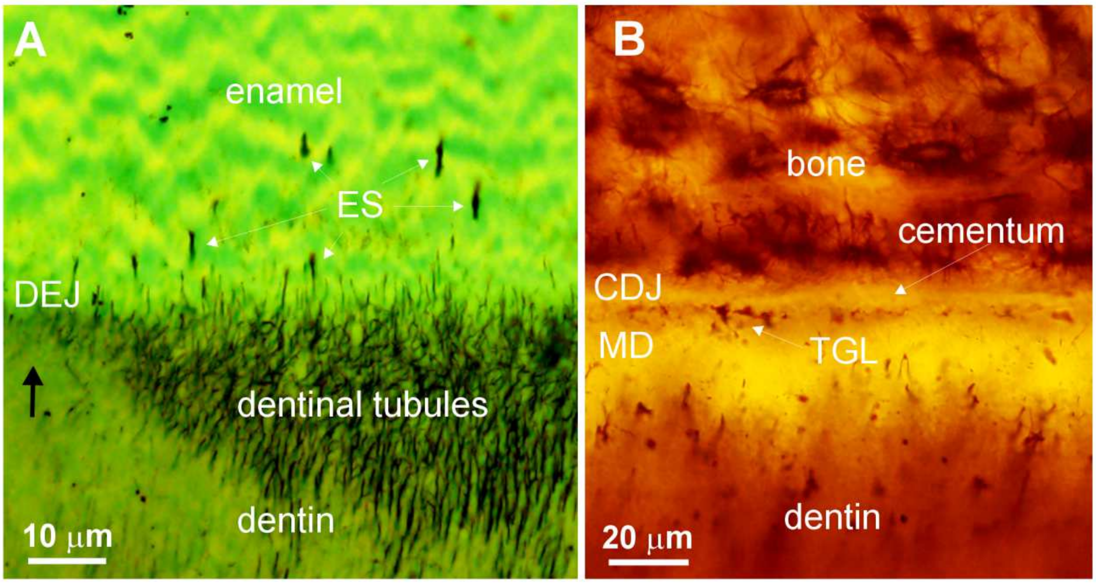

Figure S4. Compositional differences between the dentinoenamel junction (DEJ) and cementodentinal junction (CDJ) in an ornithischian tooth. (A) Two-tissue composition of an *Edmontosaurus* tooth near the DEJ observed using polarized light microscopy with a 50X objective. The wavy pattern shows the enamel region. The black arrow indicates a region that is not well preserved and has few or no dentinal tubules below the DEJ. (B) Three-tissue composition of an *Edmontosaurus* tooth near the CDJ observed using polarized light microscopy, including cementum, mantle dentin with Tome's granular layer (TGL), and bulk dentin.

Figure S4 shows the compositional differences between the dentinoenamel junction (DEJ) and cementodentinal junction (CDJ) in an ornithischian tooth. High-density dentinal tubules and enamel spindles can be observed close to the DEJ. From a structural perspective, no mantle dentin can be observed beneath the enamel (Fig. S4A). However, we can observe optical mantle dentin with Tome's granular layer between the cementum and bulk dentin in the same tooth. This implies that mantle dentin disappearing from the DEJ is one of the most important differentiation differences between Saurischia and Ornithischia.

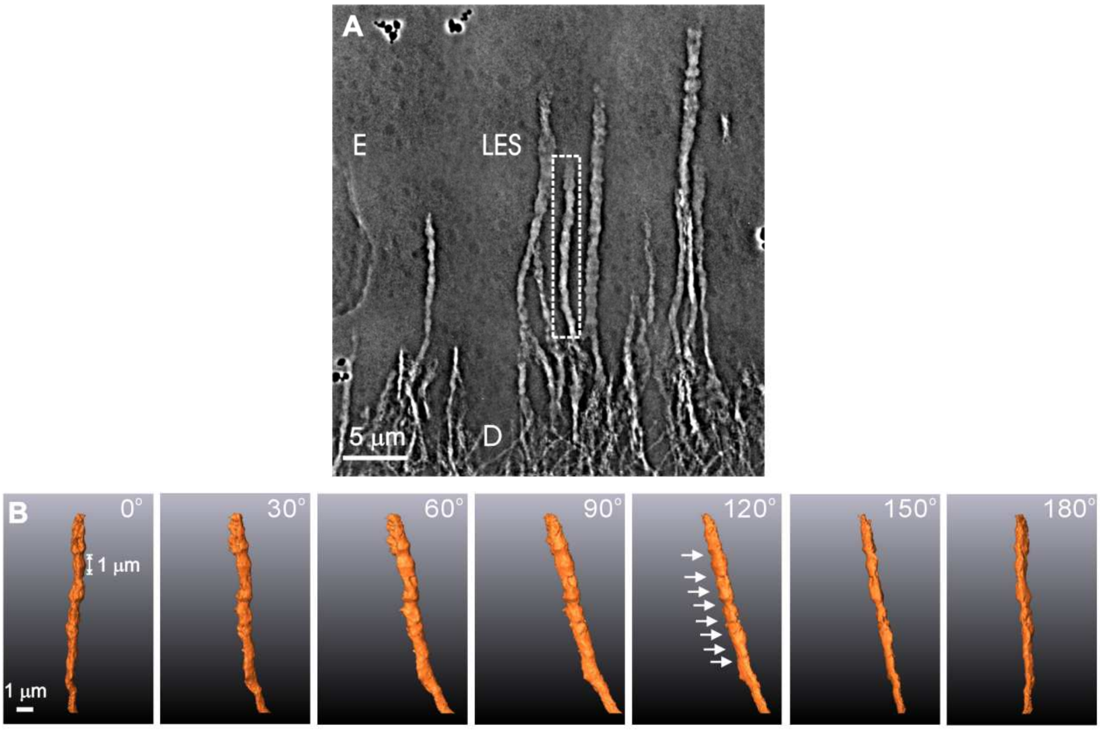

Figure S5. Periodic ES structures inside *Shantungosaurus* teeth. (A) An ultrahigh-resolution 2D TXM image of internal structures near the DEJ. (B) Corresponding ultrahigh-resolution 3D tomography inside the dashed rectangular region in (A). White arrows indicate thin regions of the long enamel spindle (LES). (E: enamel, D: dentine.)

Figure S5 shows the typical periodic LES structures observed in some ornithischian dinosaurs, such as *Shantungosaurus*. Figure S5A shows an ultrahigh-resolution 2D TXM image of the internal structures of a *Shantungosaurus* tooth near the DEJ. This figure clearly shows the periodic characteristic of the LES. Figure S5B shows the 3D tomography of a periodic LES structure from the dashed rectangular region in Figure S5A. The period of this feature is approximately 1 µm.

Clades	Character scores
Yunnanosaurus	0000--111
Diplodocus	2110--121
Dilophosaurus	2010--121
Spinosaurus	3110--131
Carcharodontosaurus	1010--111
Dromaeosaurus	0010--111
Tarbosaurus	0110--111
Tyrannosaurus	2110--131
Edmontosaurus	2001100-0
Shantungosaurus	2001110-0
Saurolophus	1101000-0
Pachycephalosaurus	1001000-0
Triceratops	2001010-0

Table S1. Internal character scores of various dinosaur teeth for cladistic analysis.

1. Thickness of enamel: (0) less than 50 micrometers; (1) 50-100 micrometers, (2) 101-200 micrometers, (3) greater than 201 micrometers.
2. Enamel crack: (0) absent; (1) present.
3. Enamel tufts: (0) absent; (1) present.
4. Enamel spindle: (0) absent; (1) present.
5. Length of long enamel spindle: (0) less than 25 micrometers; (1) more than 25 micrometers.
6. Periodic enamel spindle: (0) absent; (1) present.
7. Mantle dentin between enamel and dentin: (0) absent; (1) present.
8. Thickness of mantle dentin between enamel and dentin: (0) less than 20 micrometers; (1) 20-34 micrometers, (2) 35-42 micrometers, (3) greater than 43 micrometers.
9. Interglobular porous space between enamel and dentin: (0) absent; (1) present.
